# Accurate multiple network alignment through context-sensitive random walk

**DOI:** 10.1186/1752-0509-9-S1-S7

**Published:** 2015-01-21

**Authors:** Hyundoo Jeong, Byung-Jun Yoon

**Affiliations:** 1 Department of Electrical and Computer Engineering, Texas A&M University, College Station, USA; 2 College of Science, Engineering, and Technology, Hamad Bin Khalifa University (HBKU), Doha, Qatar

## Abstract

**Background:**

Comparative network analysis can provide an effective means of analyzing large-scale biological networks and gaining novel insights into their structure and organization. Global network alignment aims to predict the best overall mapping between a given set of biological networks, thereby identifying important similarities as well as differences among the networks. It has been shown that network alignment methods can be used to detect pathways or network modules that are conserved across different networks. Until now, a number of network alignment algorithms have been proposed based on different formulations and approaches, many of them focusing on pairwise alignment.

**Results:**

In this work, we propose a novel multiple network alignment algorithm based on a context-sensitive random walk model. The random walker employed in the proposed algorithm switches between two different modes, namely, an individual walk on a single network and a simultaneous walk on two networks. The switching decision is made in a context-sensitive manner by examining the current neighborhood, which is effective for quantitatively estimating the degree of correspondence between nodes that belong to different networks, in a manner that sensibly integrates node similarity and topological similarity. The resulting node correspondence scores are then used to predict the maximum expected accuracy (MEA) alignment of the given networks.

**Conclusions:**

Performance evaluation based on synthetic networks as well as real protein-protein interaction networks shows that the proposed algorithm can construct more accurate multiple network alignments compared to other leading methods.

## Background

With the availability of large-scale protein-protein interactions (PPI) networks, comparative network analysis tools have been gaining increasing interest as they provide useful means of investigating the similarities and differences between different networks. As demonstrated in [1,2], PPI networks of different species embed various conserved functional modules - such as signaling pathways and protein complexes - which can be detected through network querying [3-5] and network alignment [6-14]. Comparative network analysis methods allow us to transfer existing knowledge on well-studied organism to less-studied ones and they have the potential to detect potential functional modules conserved across different organisms and species [1,2,15].

There exist several different types of comparative network analysis methods, among which global network alignment methods specifically aim to predict the best overall mapping among two or more biological networks. In order to obtain biologically meaningful results, where functionally similar biomolecules across networks are accurately mapped to each other, we should consider both the molecule-level similarity between the individual molecules as well as the similarity between their interaction patterns. The former is often called the "node similarity" while the latter is typically referred to as the "topological similarity." Examination of conserved functional modules shows that many of the molecular interactions in such modules are also well conserved, clearly showing the importance of taking the topological similarity into account when comparatively analyzing biological networks. Biological networks, such as PPI networks, are typically represented as graphs, where the nodes represent individual biomolecules (e.g., proteins) and interactions (e.g., protein binding) between biomolecules are represented by edges connecting the corresponding nodes. Given these graph representations of biological networks, the network alignment problem can be formulated as an optimization problem whose goal is to find the optimal mapping - either one-to-one or many-to-many - among a set of graphs that maximizes a scoring function that assesses the goodness of a given mapping. This is essentially a combinatorial optimization problem with a exponentially large search space, which makes finding the optimal mapping practically infeasible for large networks. As a result, existing network alignment methods employ various heuristic techniques to make the network alignment problem computationally tractable.

Several network alignment algorithms have been proposed so far [6-14], many of which focus on pairwise network alignment [16]. For example, GRAAL [9] analyzes the graphlet degree signature for two PPI networks, where it can generalize the degree of node by counting the number of graphlets for each node, and then align the two networks using a seed-and-extend approach. MI-GRAAL [10] extends GRAAL by integrating further sources of information (e.g., clustering coefficient or functional similarity) to measure the similarity between two networks. PINALOG [11] is another example of pairwise network alignment algorithm, which constructs the initial mapping for protein nodes that form dense subgraphs in the respective networks. This initial mapping is further extended by subsequently finding similar nodes in the neighborhood. Recently, a number of multiple network alignment algorithms have been proposed [12-14]. For example, SMETANA [12] tries to estimate probabilistic node correspondence scores using a semi-Markov random walk model, and then uses the estimated scores to predict the maximum expected accuracy (MEA) alignment of the given networks. Given a set of networks, NetCoffee [13] generates all possible combinations of bipartite graphs for these networks, and updates the edges in each bipartite graph based on the sequence similarity of the proteins and the topological structure of the networks. Then, the algorithm finds candidate edges (i.e., mappings) in the bipartite graphs and combines qualified edges through simulated annealing. BEAMS [14] is another recent multiple network alignment algorithm, which first extracts the so-called "backbones", or the minimal set of disjoint cliques in the filtered similarity graph, and then iteratively merges these backbones to maximize the overall alignment score.

In this paper, we propose a novel multiple network alignment algorithm based on a context-sensitive random walk (CSRW) model. The employed CSRW model adaptively switches between different modes of random walk in a context-sensitive manner by sensing and analyzing the present neighborhood of the random walker. This context-sensitive behavior improves the quantitative estimation of the potential correspondence between nodes belonging to different networks, ultimately, improving the overall accuracy of the multiple network alignment as we will demonstrate through extensive performance evaluation based on real and synthetic biological networks.

## Methods

### Maximum expected accuracy (MEA) alignment of biological networks

Let us assume that we have a set of *N *PPI networks $G=\left\{G_{1},G_{2},\ldots ,G_{N}\right\}$. Each network $G_{n}=\left(V_{n},E_{n}\right)$ has a set of nodes $V_{n}=\left\{v_{1},v_{2},\ldots \right\}$ and edges $E_{n}=\left\{e_{i,j}\right\}$, where *e_i,j _*represents the interaction between nodes *v_i _*and *v_j _*in the network $G_{n}$. For each pair of PPI networks $G_{U}=\left(U,D\right)$ and $G_{V}=\left(V,E\right)$, we denote the pairwise node similarity score for a node pair (*u_i_, v_j _*), where $u_{i}\in U$ and $v_{j}\in V$, as *s*(*u_i_, v_j _*). In this study, we use the BLAST bit score between proteins as their node similarity score, but other types of similarity scores based on structural or functional similarity can be also utilized if available.

Suppose $A^{*}$ is the true alignment of the networks in the set **G**, which is unknown and needs to be predicted. As in [12,17], we can define the accuracy of a given network alignment  A as follows

(1) $$accuracy\;\left(A,A^{*}\right)=\frac{1}{\left|A\right|}\underset{u_{i}\sim v_{j}\in A}{\sum }1\left(u_{i}\sim v_{j}\in A^{*}\right),$$

where **1 **(·) is an indicator function, whose value is 1 if the mapping *u_i _*~ *v_j _*is included in the true alignment $A^{*}$ and 0 otherwise. The given measure assesses the goodness of the alignment  A based on the relative proportion of correctly aligned nodes. Of course, since the true alignment is not known, the accuracy of a network alignment  A cannot be measured using (1), hence we cannot directly use this measure to compare different potential alignments to choose the best one. A reasonable alternative would be to estimate the expected accuracy as follows

(2) $$E_{A^{*}}\left[accuracy\;\left(A,A^{*}\right)\right]=\frac{1}{\left|A\right|}\underset{u_{i}\sim v_{j}\in A}{\sum }P\left(u_{i}\sim v_{j}|G\right),$$

where *P *(*u_i _*~ *v_j_*|**G**) is the posterior alignment probability between the nodes *u_i _*and *v_j _*given the set of networks **G**. Based on this measure, our objective is then to predict the maximum expected accuracy (MEA) network alignment $\tilde{A^{*}}$ of the networks in **G **as follows

(3) $$\tilde{A^{*}}=\underset{A}{\text{max}}E_{A^{*}}\left[accuracy\;\left(A^{*},A\right)\right].$$

A similar MEA approach [18] has been formerly adopted by a number of multiple sequence alignment algorithms, including ProbCons [17], ProbAlign [19], and PicXAA [20-22]. The MEA framework has been shown to be very effective in constructing accurate alignment of multiple biological sequences, making it one of the most popular approaches for sequence alignment. Recently, the MEA approach has been also applied to comparative network analysis, where RESQUE [4] performs MEA-based network querying and SMETANA [12] performs MEA-based multiple network alignment.

### Comparing and aligning networks based on context-sensitive random walk

In order to find the alignment that maximizes the expected accuracy defined in (2), we first need an accurate method for estimating the posterior node alignment probability *P *(*u_i _*~ *v_j _|***G**). For this purpose, we adopt a context-sensitive random walk (CSRW) model, motivated by the pair hidden Markov model (pair-HMM) that has been widely used in sequence alignment [23]. The pair-HMM provides a simple, yet very effective, mathematical framework for estimating the alignment probability between symbols in different biological sequences. Unlike the traditional HMM, which generates a single symbol sequence, the pair-HMM generates a pair of aligned symbol sequences. Pair-HMM makes transitions between three different internal states *M*, *I_X _*, and *I_Y _*, where the *M *state emits an aligned pair of symbols, one symbol in sequence *X *and the other in sequence *Y*, while *I_X _*and *I_Y _*emit an unaligned symbol in sequence *X *and sequence *Y*, respectively. Given two biological sequences, the pair-HMM can be used to estimate the probability whether a given symbol pair was jointly emitted at state *M*, hence should be aligned to each other. This probability can be computed using the forward and backward algorithms and the resulting alignment probability provides us with a measure of confidence about the (biological) relevance between the given symbols (i.e., nucleotides, amino acids).

One of the most important features of pair-HMM is that it properly recognizes that conserved sequence patterns and motifs in different species may contain inserted and/or deleted symbols (often referred to as "indels") and therefore it specifically tries to model these indels. In a similar manner, a mathematical model that can recognize node insertions and deletions in different biological networks that contain conserved subnetwork regions and network motifs may be useful for obtaining a reliable posterior node-to-node alignment probability. Recently, random walk models have been shown to be effective for estimating the node correspondence in different networks [7,12,15] in a way that seam-lessly integrates both node similarity and topological similarity. However, the random walk models that were used in previous network alignment algorithms did not explicitly consider indels.

In this work, we adopt a novel context-sensitive random walk model that has been recently proposed to improve on existing models by taking such indels into account [24]. In a way that is conceptually similar to the pair-HMM, the CSRW has three different internal states *M*, $I_{U}$, and $I_{V}$, each of which corresponds to a different mode of random walk. At the *M *state, the random walker simultaneously moves on both networks to enter a pair of "matching" nodes. On the other hand, at the $I_{U}$ state, the random walker only moves on network $G_{U}$ to enter a potentially "inserted" node in $G_{U}$ that may not have a corresponding node in the network $G_{V}$. Similarly, at the $I_{V}$ state, the random walker only moves on $G_{V}$ to enter a potentially inserted node in $G_{V}$. Transitions between states take place in a context-sensitive manner, where the random walker examines the neighboring nodes to determine the mode of random walk. For example, if there are node pairs with significant node similarity (i.e., potential orthologous nodes) in the immediate neighborhood, the CSRW switches to the *M *state to make a simultaneous move on both networks and randomly enter one of these node pairs. Otherwise, the CSRW switches to either $I_{U}$ or $I_{V}$ and performs an individual random walk only on one of the networks. Based on this random walk model, we compute the long-run proportion of time that a given pair of nodes will be *simultaneously *visited (i.e., at the *M *state), which can be used to compute a probabilistic correspondence score between these two nodes, as we will describe in the following section.

### Estimation of node correspondence scores

Suppose we want to measure the correspondence between nodes that belong to two different networks $G_{U}=\left(U,D\right)$ and $G_{V}=\left(V,E\right)$, both of which are included in **G**, the set of PPI networks to be aligned. For every node pair (*u_i_*, *v_j_*), where $u_{i}\in U$ and $v_{j}\in V$, our goal is to quantify the level of confidence - which we refer to as the *node correspondence score *- using the CSRW model discussed earlier. For this purpose, we first construct the transition probability matrix that corresponds to the random walk. Let  M be the set of node pairs (*u_i_, v_j_*) with a positive pairwise node similarity score *s*(*u_i_, v_j_*)

(4) $$M=\left\{\left(u_{i},v_{j}\right)|s\left(u_{i},v_{j}\right)>0,u_{i}\in U,v_{j}\in V\right\}.$$

We also define the set of non-similar node pairs as follows

(5) $$I=\left\{\left(u_{i},v_{j}\right)|s\left(u_{i},v_{j}\right)=0,u_{i}\in U,v_{j}\in V\right\}.$$

Let the current position of the random walker in the product graph be (*u_c_, v_c_*), where $u_{c}\in U$ and $v_{c}\in V$. In each time step, the random walker examines the set of similar neighboring nodes $N\left(u_{c},v_{c}\right)=\left\{\left(u_{i},v_{j}\right)|u_{i}\in N\left(u_{c}\right),v_{j}\in N\left(v_{c}\right),\left(u_{i},v_{j}\right)\in M\right\}$ to determine its mode of random walk (corresponding to one of the three possible internal states), where $N\left(u_{c}\right)$ is the set of neighbors of the node *u_c _*in the network $G_{U}$ and $N\left(v_{c}\right)$ is the set of neighbors of the node *v_c _*in the network $G_{V}$. If there are similar node pairs among the neighboring node pairs, hence $N\left(u_{c},v_{c}\right)$ is not empty, the random walker switches its internal state to the *M *state and performs a simultaneous walk on both networks, moving from (*u_c_*, *v_c_*) to one of the nodes

$\left(u_{i},v_{j}\right)\in N\left(u_{c},v_{c}\right)$. We define the transition probability for this simultaneous walk as follows

(6) $$P\left[\left(u_{i},v_{j}\right)|\left(u_{c},v_{c}\right)\right]=\frac{s\left(u_{i},v_{j}\right)}{\underset{\left(u_{i^{\prime }},v_{i^{\prime }}\right)\in N\left(u_{c},v_{c}\right)}{\sum }s\left(u_{i^{\prime }},v_{i^{\prime }}\right)}.$$

In case there is no similar node pair around the current position of the random walker, that is $N\left(u_{c},v_{c}\right)=\emptyset$, the random walker randomly changes its state to either $I_{U}$ or $I_{V}$, and performs an individual walk on the corresponding network $G_{U}$ or $G_{V}$. The probability that a given network will be chosen for an individual random walk is proportional to its size (i.e., number of nodes in the network), which ensures that both networks are equally well-traversed at the *I *states. The random walker randomly moves to one of the neighboring nodes with equal probability on the selected network, while staying at the same node on the other network. Based on this behavior, the transition probabilities at state $I_{U}$ are given by

(7a) $$P\left[\left(u_{i},v_{c}\right)|\left(u_{c},v_{c}\right)\right]=\frac{\left|U\right|}{\left|U|+|V\right|}\times \frac{1}{\left|N\left(u_{c}\right)\right|}$$

for $u_{i}\in N\left(v_{c}\right)$, and the transition probabilities at state $I_{V}$ are given by

(7b) $$P\left[\left(u_{c},v_{j}\right)|\left(u_{c},v_{c}\right)\right]=\frac{\left|V\right|}{\left|U|+|V\right|}\times \frac{1}{\left|N\left(v_{c}\right)\right|},$$

for $v_{j}\in N\left(v_{c}\right)$.

Based on the transition probabilities given by (6), (7a), and (7b), we can construct the transition probability matrix **P **for the random walk on the two networks $G_{U}$ and $G_{V}$. Given **P**, we can estimate the longrun proportion of time that the random walker spends in each pair of nodes (*u_i_, v_j_*) by computing the steady state distribution *π*. In practice, since real PPI networks typically have a relatively small number of interactions (therefore only few edges for most nodes), the resulting transition probability matrix for the CSRW is sparse, which makes it relatively straightforward to compute the steady state distribution using the power method.

In order to increase the computational efficiency of the proposed network alignment method, instead of using the original transition probability matrix **P**, we use a reduced matrix $\tilde{P}$. The reduced matrix $\tilde{P}$ is obtained by removing the rows and columns in **P **that correspond to node pairs in  I while keeping only the rows and columns that correspond to node pairs in  M. After the reduction, $\tilde{P}$ is re-normalized to make it a legitimate stochastic matrix. In practice, since the CSRW is designed to spend more time at node pairs with higher similarity, the random walker spends a relatively small amount of time at node-pairs that belong to the set  I, and using the reduced matrix $\tilde{P}$ instead of **P **only minimally affects the estimated long-run proportion of time spent at $\left(u_{i},v_{j}\right)\in M$. As a result, the difference in terms of network alignment performance that results from replacing the original matrix **P **by this reduced matrix $\tilde{P}$ appears to be small as shown in the supplementary material (see Section S1).

We make one further modification to the CSRW in [24] by allowing the random walker to restart at a new position at each time step with a fixed restart probability *λ*. Note that a similar "random walk with restart" approach was used by IsoRank [6] and IsoRankN [7], although these algorithms do not utilize the CSRW adopted in our method. We allow the random walker to select its restart position according to the pairwise node similarity, such that node pairs with higher node similarity have higher chance to be the restart position of the random walker. To this aim, we normalize the pairwise node similarity scores so that they sum up to 1. Our final node correspondence score vector **c **is obtained from a linear combination of the steady-state distribution of the context-sensitive random walker $\tilde{\pi }$ (estimated using the reduced transition probability matrix $\tilde{P}$) and the normalized node similarity score vector **s **as follows

(8) $$c=\lambda s+\left(1-\lambda \right)\tilde{\pi }.$$

The above formulation, obtained by allowing the CSRW to restart the random walk at a new position, is especially useful when comparing real PPI networks, which are often incomplete and contain many isolated nodes. Simulation results show that the incorporation of the restart scheme can make our CSRW-based alignment method more robust, especially when the available topological data are either unreliable or insufficient for detecting the similarities between networks (see Section S2).

In order to determine the restart probability *λ*, we first analyze the structure of the reduced product graph of $G_{U}$ and $G_{V}$ that contains only similar node pairs included in  M. Intuitively, it is desirable to increase the restart probability *λ *if the networks are disconnected and decrease the probability if the networks are well connected. For example, if all the nodes in the reduced product graph are completely disconnected, it is desirable to restart the random walker at every step. Additionally, when we consider the following two cases - (i) most nodes in the product graph are connected and there are only a few disconnected nodes; (ii) the product graph is equally divided into *N *connected subnetworks of identical size - it would be desirable to assign a higher *λ *to the latter case. Based on these intuitions, we set the restart probability *λ *as the ratio of the total number of nodes in the top *K*% smallest subnetworks to the total number of nodes in the reduced product graph. In this work, we used *K *= 99% to determine the restart probability *λ*.

### Constructing the multiple network alignment

Once we have computed the node correspondence scores in (8) for every pair of networks in **G**, we take a greedy approach as in [12] to construct the multiple network alignment. The overall alignment process is as follows. First, in order to improve the reliability of the node correspondence scores, we selectively apply the probabilistic consistent transformation (PCT) defined in [12]. If *λ *is larger than a predefined threshold *λ_t_*, we do not apply PCT to the node correspondence scores. A large *λ *implies that the product graph is ill connected (e.g., containing a large number of isolated nodes), in which case applying the PCT would not be helpful and may in fact make the scores less reliable. This is because the PCT in [12] was developed based on the assumption that the product graphs for all network pairs are relatively well connected. After the potential score refinement step through PCT, we begin with an empty alignment and greedily add aligned node pairs (*u_i_*, *v_j_*) to the network alignment, starting from the pairs with the highest node correspondence scores, until there is no other node pair left that can be added without creating inconsistencies in the network alignment. Assuming that the node correspondence scores in (8) obtained by the context-sensitive random walk model with restart accurately reflect the true correspondence between nodes - such that the score is proportional to the posterior node alignment probability - the proposed network alignment scheme can be viewed as a heuristic way to find the MEA alignment of the networks in **G**.

## Results and discussion

### Datasets and experimental set-up

To assess the performance of the proposed method, we tested the proposed network alignment method based on PPI networks in NAPAbench [25] and IsoBase [26]. NAPAbench is a network alignment benchmark that consists of 3 different datasets, referred to as the pairwise alignment dataset, 5-way alignment dataset, and 8-way alignment dataset. Each dataset contains three different subsets of 10 network families, each subset created using a different network growth model - CG (crystal growth), DMC (duplication-mutation-complementation), and DMR (duplication with random mutation). Each network family consists of 2, 5, or 8 PPI networks depending on the alignment dataset. For network families in the pairwise alignment dataset, each family contains one network with 3,000 nodes and the other with 4,000 nodes. In the 5-way network alignment dataset, a network family consists of 5 networks with 1,000, 1,500, 2,000, 2,500, and 2,500 nodes. Finally, in the 8-way alignment dataset, every network family consists of 8 networks, where each network contains 1,000 nodes. To evaluate the performance of the proposed method on real PPI networks, we utilized IsoBase datasets [26], which was constructed by integrating the following databases: BioGRID [27], DIP [28], HPRD [29], MINT [30], and IntAct [31]. IsoBase contains the PPI networks of five species: *H. sapiens*, *M. musculus*, *D. melanogaster*, *C. elegans*, and *S. cerevisiae*. Currently, the PPI network of *H. sapiens *in [26] has 22,369 proteins and 43,757 interactions, the PPI network of *M. musculus *has 24,855 proteins and 452 interactions, the PPI network of *D. melanogaster *has 14,098 proteins and 26,726 interactions, the PPI network of *C. elegans *has 19,756 proteins and 5,853 interactions, and the PPI network of *S. cerevisiae *has 6,659 proteins and 38,109 interactions. In our analysis, we excluded the *M. musculus *network as it currently contains only a small number of interactions.

Based on our simulations, we report the following performance metrics: correct nodes (CN), specificity (SPE), mean normalized entropy (MNE), conserved interaction (CI), coverage, and computation time. CN is the total number of nodes in the correct equivalence classes. Given a network alignment, an equivalence class is defined as the set of aligned nodes, and if all nodes in the equivalence class have the same functionality the given equivalence class is said to be correct. SPE is the relative number of correct equivalence classes to the total number of equivalence classes in a network alignment. For each equivalence class **C**, the normalized entropy can be computed by $H\left(\text{C}\right)=-\frac{1}{\text{log}\;d}\sum_{i=1}^{d}p_{i}\;\text{log}\;p_{i}$, where *p_i _*is the relative proportion of nodes in **C **with functionality *i *and *d *is the total number of different functionalities in the given equivalence class. As a result, a network alignment that accurately maps functionally similar nodes, hence being functionally consistent, will have lower mean normalized entropy. CI is defined as the total number of edges between equivalence classes. We also count the total number of edges between correct equivalence classes, which we refer to as the conserved orthologous interactions (COI), to assess the biological relevance of the conserved interactions that have been identified by the network alignment method. Finally, for 5-way and 8-way alignment datasets, we measure the equivalence class coverage and the node coverage, where the former is the number of equivalence classes that include nodes from *k *different networks, and the latter is the number of nodes in an equivalence class whose equivalence class coverage is *k*. For the performance evaluation based on real PPI networks in IsoBase, we determined the functionality of each protein using the KEGG protein annotation [32,33]. Note that nodes without any functional annotation in each equivalence class and equivalence classes that consist of a single node or nodes from a single network were removed before computing the performance metrics.

We compared the performance of the proposed multiple network alignment method against a number of state-of-the-art algorithms: SMETANA [12], PINALOG [11], BEAMS [14], NetCoffee [13], and IsoRankN [7]. NetCoffee was not included in pairwise network alignment experiments, since it requires at least 3 networks. For multiple network alignment experiments, PINALOG was excluded as the algorithm can only handle pairwise alignments. For IsoRankN, we set the parameter *α *to 0.6 as in the original paper [7]. For BEAMS, we set the filtering threshold to 0.4 for IsoBase and 0.2 for NAPAbench as in the original paper [14], and set the parameter *α *to 0.5. The parameter *α *for NetCoffee was set to 0.5. We used default parameters for SMETANA (i.e., *n*_max _= 10, *α *= 0.9, and *β *= 0.8), and the same parameters were used in the proposed network alignment method as well. Finally, in the proposed method, we used *λ_t _*= 0.7 to determine whether or not to apply PCT to the estimated node correspondence scores.

All experiments were performed on a personal computer with a 2.4 GHz Intel i7 processor and 8 GB memory.

### Performance assessment based on NAPAbench network alignment benchmark

We first evaluated the performance of the proposed algorithm using the NAPAbench network alignment benchmark and compared it to other leading algorithms. The evaluation results are summarized in Table 1, 2, and 3, which show the average CN, SPE, and MNE of various network alignment algorithms.

**Table 1:** Performance comparison for pairwise network alignment.

	DMC			DMR			CG		
			
	CN	SPE	MNE	CN	SPE	MNE	CN	SPE	MNE
Proposed	**5,593.9**	**0.958**	**0.039**	**5,305.3**	**0.939**	**0.055**	4,893.2	0.942	0.054

SMETANA	5,164.5	0.926	0.068	4,900.6	0.916	0.078	4,846.2	**0.949**	**0.048**

BEAMS	5,076.5	0.826	0.150	5,176.7	0.840	0.138	**5,441.2**	0.870	0.112

PINALOG	3,779	0.726	0.274	3,533.4	0.683	0.317	4,325	0.788	0.212

IsoRankN	3,816.5	0.827	0.163	3,905.2	0.836	0.155	3,863.2	0.832	0.159

**Table 2:** Performance comparison for 5-way network alignment.

	DMC			DMR			CG		
			
	CN	SPE	MNE	CN	SPE	MNE	CN	SPE	MNE
Proposed	**7,536.7**	**0.940**	**0.047**	**7,410.3**	**0.934**	**0.053**	7,177.6	0.919	0.060

SMETANA	7,273.2	0.912	0.069	7,181.8	0.915	0.068	7,331.6	**0.935**	**0.048**

BEAMS	6,842.2	0.863	0.104	6,882	0.873	0.096	**7,376.5**	0.921	0.062

NetCoffee	6,431.2	0.894	0.090	6,395.7	0.890	0.093	6,150.2	0.854	0.120

IsoRankN	5,559	0.920	0.147	5,462.3	0.793	0.162	5,688.4	0.828	0.132

Proposed (all 5 species)	**4476.9**	**0.931**	**0.048**	**4017.9**	**0.916**	**0.060**	3644.8	0.900	0.068

SMETANA (all 5 species)	4062.3	0.891	0.077	3704.9	0.889	0.080	**3778.9**	**0.922**	**0.052**

BEAMS (all 5 species)	2858.4	0.814	0.121	3095.2	0.838	0.104	3510.3	0.918	0.052

NetCoffee (all 5 species)	2960.4	0.867	0.106	2973.3	0.855	0.113	2841.2	0.796	0.156

IsoRankN (all 5 species)	1668.1	0.728	0.179	1595.4	0.677	0.215	2233.5	0.742	0.168

**Table 3:** Performance comparison for 8-way network alignment.

	DMC			DMR			CG		
			
	CN	SPE	MNE	CN	SPE	MNE	CN	SPE	MNE
Proposed	**6,621.3**	**0.901**	**0.080**	**6,467.2**	**0.891**	**0.090**	6,345.4	0.884	0.090

SMETANA	6,336.7	0.869	0.106	6,195.2	0.860	0.114	6,481.2	**0.897**	**0.079**

BEAMS	6,083.1	0.825	0.163	6,063.5	0.826	0.162	**6,537.6**	0.877	0.111

NetCoffee	5,127.2	0.757	0.206	5,084.1	0.750	0.213	4,944.1	0.724	0.239

IsoRankN	4,069.1	0.644	0.268	3,916.7	0.623	0.284	3,860	0.612	0.291

Proposed (all 8 species)	**4116**	**0.961**	**0.034**	**3473.7**	**0.930**	**0.059**	3689.5	0.945	0.043

SMETANA (all 8 species)	3686.7	0.920	0.066	3348.9	0.907	0.075	**3785.6**	**0.960**	**0.031**

BEAMS (all 8 species)	2897.9	0.905	0.095	3054.7	0.901	0.099	3475.1	0.989	0.011

NetCoffee (all 8 species)	3300.8	0.837	0.136	3331.8	0.822	0.148	3317.8	0.800	0.172

IsoRankN (all 8 species)	2002.8	0.569	0.284	1775.8	0.542	0.303	2161.6	0.536	0.303

As we can see in Table 1 in most cases, the proposed algorithm yields a significantly higher CN and SPE compared to other algorithms, which shows that the algorithm is capable of finding conserved nodes with both high sensitivity and specificity. Furthermore, the mean normalized entropy (MNE) is also much lower, indicating that the proposed algorithm yields network alignment results that are more functionally coherent. This table shows that BEAMS yields higher CN for the CG dataset, although its SPE is lower and its MNE is higher than the proposed method. Both SMETANA and the proposed algorithm shows similar performance on the CG dataset, but we can also see that the proposed algorithm consistently outperforms SMETANA on the DMC/DMR datasets. Multiple network alignment results obtained using the 5-way alignment dataset and the 8-way alignment dataset show similar trends. Tables 2 and 3 show that, in most cases, our proposed algorithm outperforms other algorithms with higher CN, higher SPE, and lower MNE. For multiple network alignment, we further compared different network alignment algorithms based on their capability of predicting equivalence classes that span all networks, since one of the main goals of multiple network alignment is to find functionally homologous proteins that are conserved in the networks of all target species. Simulation results show that, in most cases, our proposed method also yields much higher CN and SPE as well as lower MNE for equivalence classes that span all networks.

**Figure 1:**
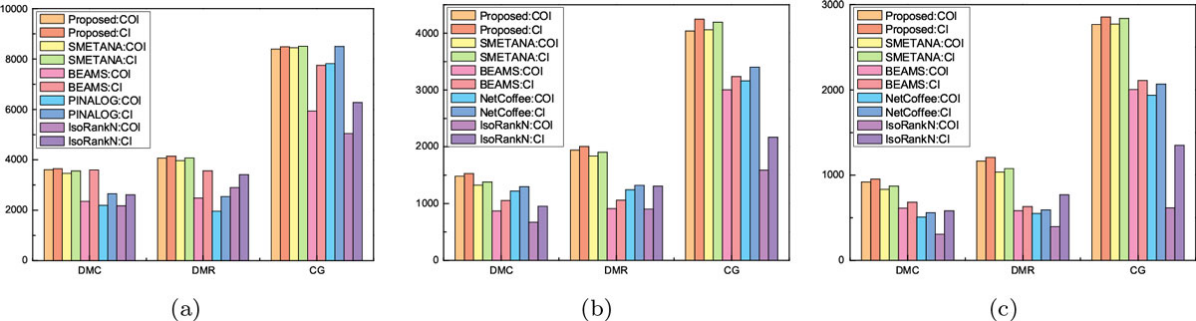
**The total number of conserved orthologous interactions (COI) and conserved interactions (CI): (a) pairwise network alignment; (b) 5-way network alignment; (c) 8-way network alignment**.

Next, we compare the number of conserved (orthologous) interactions identified by different network alignment algorithms. As Figure 1 shows, the proposed method was able to identify the largest number of conserved interactions as well as conserved orthologous interactions in most cases, resulting in higher CI and COI. The performance of SMETANA was comparable to the proposed method, while other algorithms typically resulted in lower CI and COI. It is worth noting that more than 95% of the conserved interactions that were detected by our proposed network alignment algorithm were between correct equivalence classes (i.e., conserved orthologous interactions). This certainly shows that our method can effectively detect biologically meaningful conserved interactions through network alignment.

**Figure 2:**
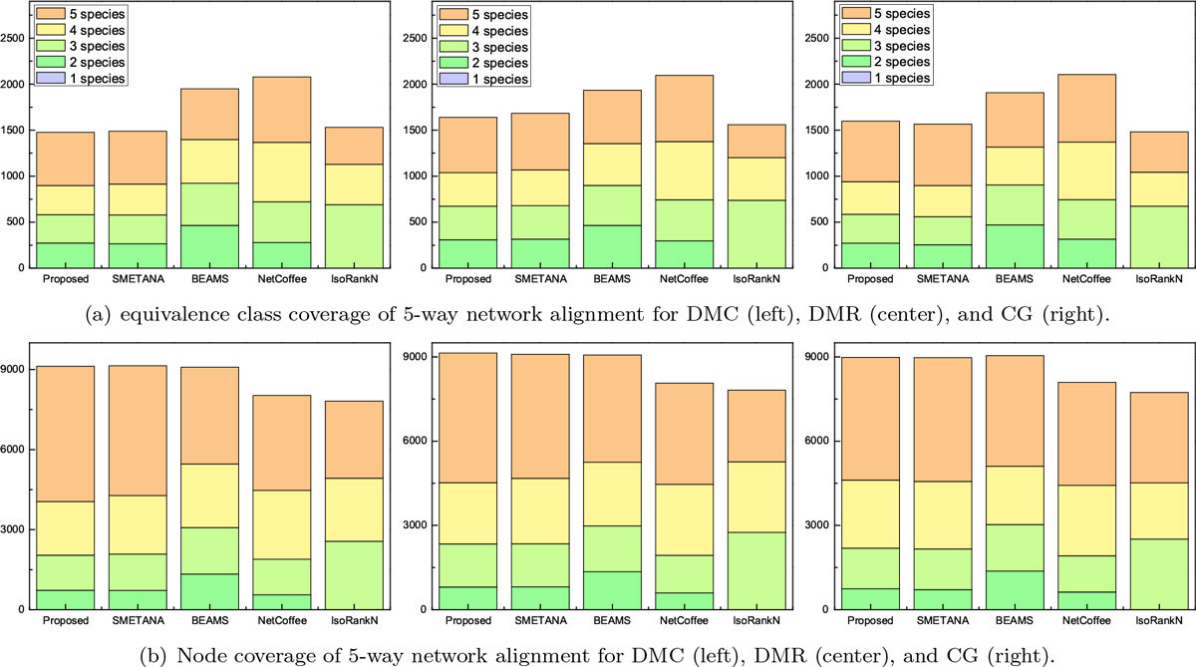
**Equivalence class coverage and node coverage for 5-way network alignment: (a) equivalence class coverage for each network growth model; (b) node coverage for each network growth model**.

**Figure 3:**
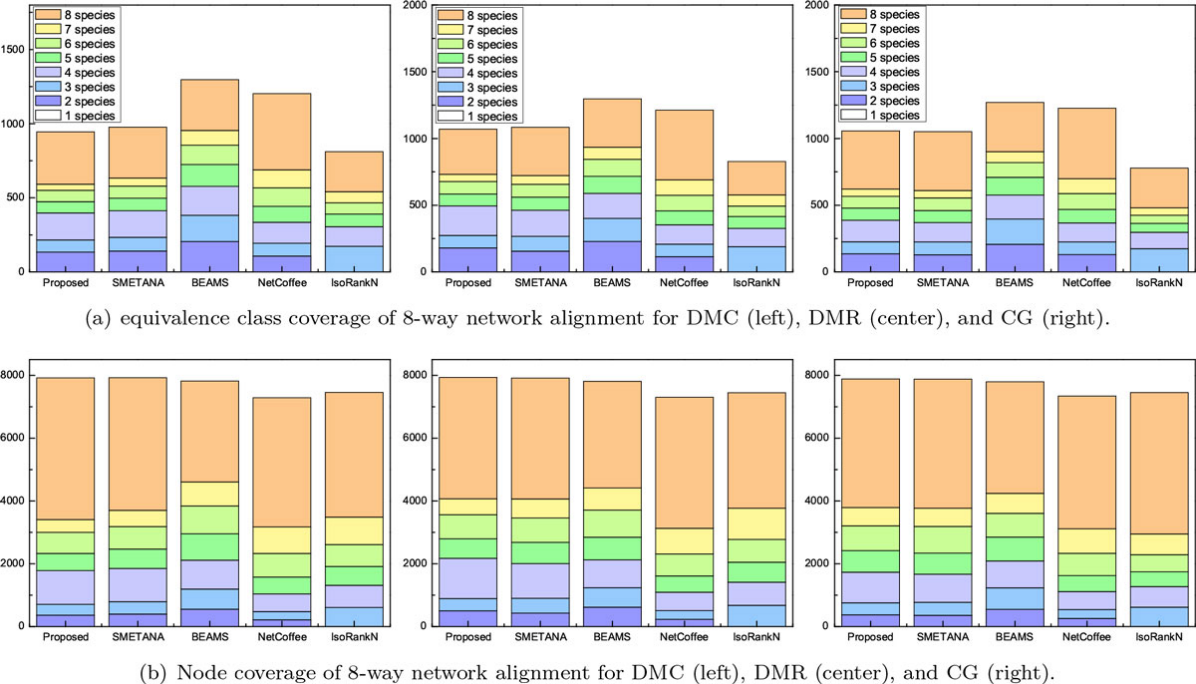
**Equivalence class coverage and node coverage for 8-way network alignment: (a) equivalence class coverage for each network growth model; (b) node coverage for each network growth model**.

We also analyzed the overall coverage of the predicted alignment results for the 5-way and 8-way network alignments. The results are shown in Figure 2 for the 5-way alignment and in Figure 3 for the 8-way alignment. For the 5-way network alignment, we can see that around 40% of the equivalence classes predicted by the proposed method contained nodes from all 5 networks. SMETANA shows a similar level of coverage, while for the remaining algorithms, only about 30% of the predicted equivalence classes included nodes from all 5 networks. The overall node coverage also shows similar trends. The 8-way alignment results summarized in Figure 3 show that the proposed algorithm can effectively find equivalence classes with good coverage, which include nodes from a large number of networks. For example, we can see that around 40% of the equivalence classes predicted by the proposed method contained nodes from all 8 networks.

Table 4 shows the mean computation time of the respective algorithms for aligning the network families in the NAPAbench datasets. As we can see in Table 4 SMETANA requires the least amount of time for aligning the networks in NAPAbench, while IsoRankN needs the most computation time. In our simulations, we observed that NetCoffee runs relatively fast, although its computation time varies significantly depending on the network structure. For example, it took much longer to align networks in the DMR dataset using NetCoffe, compared to networks in the DMC or CG datasets.

**Table 4:** Mean computation time for aligning PPI networks in the NAPAbench datasets (in seconds).

Algorithms	Pairwise	5-way	8-way	Average
Proposed	117.8	273.1	178.7	189.8

SMETANA	6.9	58.0	70.7	45.2

BEAMS	42.4	134.8	333.8	170.3

PINALOG	77.1	·	·	77.1

NetCoffee	·	132.7	225.7	179.2

IsoRankN	1083.7	3326.1	2694.8	2368.2

### Performance assessment based on protein-protein interaction networks in IsoBase

For further evaluation, we performed additional experiments using real PPI networks in IsoBase. Table 5 shows the pairwise network alignment performance of the tested algorithms for several PPI network pairs. As we can see in this table, the proposed algorithm consistently performs fairly well in all cases, outperforming the other algorithms. We can make similar observations in Table 6 which summarizes the performance evaluation results for aligning 3 PPI networks. The proposed algorithm attains high CN, high SPE, and low MNE across all cases, showing that it can effectively compare and accurately align real PPI networks. BEAMS shows good performance on multiple alignment of real networks that is comparable to the proposed method, with a slightly lower SPE and a slightly higher MNE. Additionally, although BEAMS and IsoRankN achieve higher CN in some cases, the proposed method consistently yields higher CN than these methods with comparable SPE and MNE when we consider multiple network alignment results for regions that are conserved across all networks. Another observation we can make in Table 5 is that IsoRankN performs very well on real PPI networks compared to the other more recent algorithms. This is especially interesting, if we consider the fact that the performance of IsoRankN lagged behind the other algorithms according to the large-scale evaluations using NAPAbench. One possible explanation is that, for constructing the network alignment, IsoRankN relies on node similarity (i.e., sequence similarity in this case) more strongly compared to the other algorithms. In order to find out whether this is indeed a plausible explanation, we performed network alignment experiments solely using node similarity scores (i.e., without considering network topology), where we constructed the network alignment in a greedy manner by iteratively adding protein pairs with the highest node similarity scores. The alignment results are shown in Tables 5 and 6 right below the results for IsoRankN (labeled as "Node Similarity"). Surprisingly, these results show that this simple greedy network alignment approach that uses node similarity alone outperforms IsoRankN in most cases and surpasses all the other algorithms in all cases. In fact, currently available PPI networks are known to be very incomplete and these network typically contain a large number of isolated nodes. They are suspected to include a large number of spurious interactions while still missing many potential protein-protein interactions [34,35]. Furthermore, only a small proportion of proteins in these PPI networks have reliable functional annotations (e.g., according to KEGG orthology), making it difficult to reliably assess the quality of a predicted network alignment. As a result, for current PPI networks, utilization of topological similarity between networks may not be necessarily helpful for improving the overall quality of the network alignment across the entire network. Moreover, since only a few large real PPI networks are available at the moment, we risk overtraining network alignment algorithms if they are mainly evaluated solely based on real PPI networks.

**Table 5:** Pairwise network alignment results for real PPI networks.

	H.sa-S.ce			D.me-S.ce			C.el-S.ce		
	**CN**	**SPE**	**MNE**	**CN**	**SPE**	**MNE**	**CN**	**SPE**	**MNE**

Proposed	1307	0.689	0.310	1725	0.727	0.277	1543	0.796	0.196

SMETANA	1190	0.671	0.331	1579	0.709	0.295	1443	0.771	0.222

BEAMS	1306	0.649	0.347	1636	0.675	0.320	1499	0.742	0.247

PINALOG	1100	0.682	0.324	1368	0.722	0.289	640	0.737	0.266

IsoRankN	1367	**0.765**	**0.238**	1641	0.777	0.230	1458	**0.843**	**0.155**

Node Similarity	**1486**	0.740	0.259	**1832**	**0.779**	**0.224**	**1670**	0.831	0.163

	**D.me-H.sa**			**D.me-C.el**			**C.el-H.sa**		

	**CN**	**SPE**	**MNE**	**CN**	**SPE**	**MNE**	**CN**	**SPE**	**MNE**

Proposed	2681	0.724	0.279	2714	0.855	0.146	1995	0.771	0.224

SMETANA	2274	0.671	0.331	2458	0.827	0.175	1684	0.737	0.255

BEAMS	2612	0.658	0.338	2738	0.808	0.192	1941	0.691	0.300

PINALOG	1172	0.604	0.412	672	0.689	0.317	482	0.677	0.325

IsoRankN	2635	**0.759**	**0.246**	2488	0.851	0.150	1881	**0.783**	**0.216**

Node Similarity	**2932**	0.750	0.251	**2897**	**0.875**	**0.125**	**2185**	0.770	0.227

**Table 6:** Multiple network alignment results for real PPI networks (for 3 species).

	D.me-C.el-H.sa			S.ce-C.el-H.sa			S.ce-D.me-C.el			S.ce-D.me-H.sa		
	**CN**	**SPE**	**MNE**	**CN**	**SPE**	**MNE**	**CN**	**SPE**	**MNE**	**CN**	**SPE**	**MNE**

Proposed	4331	0.705	0.289	3077	0.709	0.281	3581	0.746	0.247	3637	0.672	0.326

SMETANA	3871	0.663	0.331	2625	0.657	0.333	3227	0.714	0.279	3108	0.616	0.380

BEAMS	4354	0.676	0.316	3084	0.671	0.320	3606	0.727	0.267	3629	0.627	0.366

NetCoffee	1471	0.552	0.451	1234	0.575	0.426	1477	0.593	0.414	1877	0.540	0.465

IsoRankN	4423	0.717	0.279	3131	0.711	0.282	3464	0.749	0.245	3752	0.684	0.313

NodeSimilarity	**4775**	**0.746**	**0.248**	**3457**	**0.737**	**0.256**	**3920**	**0.798**	**0.197**	**4132**	**0.719**	**0.278**

Proposed (all 3-species)	**3926**	0.702	0.290	**2387**	0.724	0.265	**2624**	0.715	0.271	**2540**	0.681	0.315

SMETANA (all 3-species)	3442	0.671	0.323	2106	0.677	0.312	2378	0.685	0.301	2225	0.630	0.363

BEAMS (all 3-species)	3867	0.687	0.304	2277	0.711	0.278	2573	0.718	0.272	2441	0.672	0.318

NetCoffee (all 3-species)	747	0.518	0.478	578	0.528	0.465	713	0.538	0.462	1167	0.516	0.489

IsoRankN (all 3-species)	3757	**0.753**	**0.241**	2323	**0.775**	**0.215**	2470	**0.732**	**0.258**	2510	**0.726**	**0.267**

**Figure 4:**
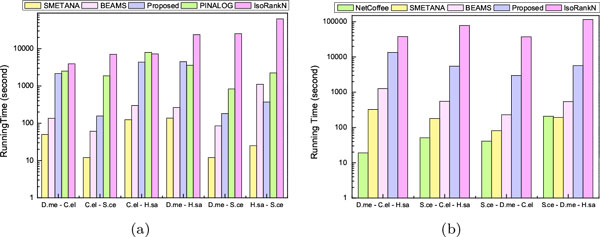
**Computation time for aligning real PPI networks (in seconds): (a) pairwise network alignment; (b) multiple network alignment (for 3 species)**.

Figure 4 shows the computation time for aligning the PPI networks in IsoBase. SMETANA required the least computation time for pairwise network alignment and NetCoffee was the fastest among all for aligning the PPI networks of 3 species. Although IsoRankN yielded accurate alignment results for real PPI networks in IsoBase, it also required the largest amount of computation time in most cases. Figure 4 shows that our proposed network alignment algorithm requires relatively longer running time compared to other algorithms, in exchange for the improved alignment accuracy. Currently, the main bottleneck is the time required to construct the transition probability matrix $\tilde{P}$ of the context-sensitive random walker, and we are currently optimizing the code for our algorithm to make it computationally more efficient.

## Conclusions

In this paper, we proposed a novel network alignment algorithm based on a context-sensitive random walk model that has been recently introduced. The CSRW provides an effective mathematical framework for comparing different biological networks and quantifying the node-to-node correspondence between nodes that belong to different networks. In our proposed method, we combined the CSRW model with a restart scheme, where the restart probability is automatically adjusted based on the characteristics of the networks under comparison. Furthermore, the proposed network alignment algorithm employs adaptive probabilistic consistency transformation, where the PCT is adaptively activated or deactivated based on the overall structure of the given networks. As we have shown through extensive performance evaluations based on biologically realistic PPI networks in NAPAbench as well as real PPI networks in IsoBase, the novel network alignment algorithm proposed in this paper can significantly improve the overall accuracy of pairwise as well as multiple network alignment.

## Competing interests

The authors declare that they have no competing interests.

## Authors' contributions

Conceived the method: HJ, BJY. Developed the algorithm and performed the simulations: HJ. Analyzed the results and wrote the paper: HJ, BJY.
